# Liposome Encapsulation Enhances Ripasudil Therapeutic Efficacy Against Proliferative Vitreoretinal Diseases: Implications in Advanced Ocular Treatment

**DOI:** 10.1167/iovs.66.6.56

**Published:** 2025-06-17

**Authors:** Rui Ji, Keijiro Ishikawa, Wei Tan, Kenichiro Mori, Ryotaro Tsukamoto, Naoya Matsunaga, Kohei Kiyohara, Yosuke Fukuda, Iori Wada, Tomoyuki Isobe, Tomohito Tanihara, Yuya Yoshida, Kouta Mayanagi, Kosuke Oyama, Yuma Terada, Kaita Otsuki, Kengo Hamamura, Hiroshi Kikuchi, Shintaro Nakao, Shigeo Yoshida, Ram Kannan, Shigehiro Ohdo, Koh-Hei Sonoda

**Affiliations:** 1 Department of Ophthalmology, Graduate School of Medical Sciences, Kyushu University, Fukuoka, Japan; 2 Department of Clinical Pharmacokinetics, Faculty of Pharmaceutical Sciences, Kyushu University, Fukuoka, Japan; 3 Tokyo New Drug Research Laboratories, Kowa Company, Ltd., Tokyo, Japan; 4 Department of Drug Discovery Structural Analysis, Faculty of Pharmaceutical Sciences, Kyushu University, Fukuoka, Japan; 5 DDS Strategy Firm, Tokyo, Japan; 6 Department of Ophthalmology, Juntendo University School of Medicine, Tokyo, Japan; 7 Department of Ophthalmology, Kurume University School of Medicine, Fukuoka, Japan; 8 Doheny Eye Institute, Pasadena, California, United States; 9 Department of Pharmaceutics, Faculty of Pharmaceutical Sciences, Kyushu University, Fukuoka, Japan

**Keywords:** proliferative vitreoretinopathy (PVR), fibrosis, age-related macular degeneration (AMD)

## Abstract

**Purpose:**

Proliferative vitreoretinal diseases, such as proliferative vitreoretinopathy (PVR) and neovascular age-related macular degeneration (nAMD), pose substantial challenges in their advanced stages owing to the development of retinal fibrous membranes. Current therapeutic modalities, including surgical interventions for PVR and antivascular endothelial growth factor therapy for nAMD, cannot effectively manage intraocular fibrosis associated with epithelial-to-mesenchymal transition (EMT) in retinal pigment epithelium (RPE) cells. Through drug screening, we identified ripasudil, a Rho-kinase inhibitor, as a remarkable suppressor of RPE-EMT. However, the short vitreal half-lives of small-molecule drugs, coupled with the limited stability of ripasudil in the ocular environment, impede its application in vitreoretinal diseases. Considering the advances in nanotechnology-assisted improvement in drug stability and cellular uptake as well as controlled release, we aimed to enhance the efficacy of ripasudil through liposome encapsulation.

**Methods:**

After ripasudil encapsulation, we performed comprehensive in vivo and in vitro analyses and pharmacokinetic studies.

**Results:**

Liposome-encapsulated ripasudil (Lipo-Ripa) demonstrated a substantial reduction in subretinal fibrosis in an advanced AMD model and more effective inhibition of PVR progression in rabbits than that induced by ripasudil alone. Pharmacokinetic studies revealed that Lipo-Ripa exhibited improved retention capacity in the vitreous and retina, alongside reduced permeability through the RPE barrier and increased cellular uptake. These characteristics resulted in a sustained elevation of drug concentration within the ocular tissues over time.

**Conclusions:**

Our findings suggest that liposomal encapsulation of ripasudil supports enhanced bioavailability and effectiveness of the drug, presenting a promising innovative therapeutic approach for the treatment of proliferative vitreoretinopathy.

Proliferative vitreoretinal diseases, including proliferative vitreoretinopathy (PVR) and neovascular age-related macular degeneration (nAMD), are the leading causes of severe vision impairment and blindness.^1^^,^^2^ These diseases are functionally characterized by the formation of retinal fibrous membranes, which may occur both above and beneath the retina. They are associated with abnormal vascular growth, the proliferation of fibrous tissues, or both, in the retinal layer.^3^^,^^4^ This pathological process fundamentally distorts the retinal architecture and influences its functions. Currently, the standard treatment for PVR involves surgery, which is potentially associated with complications leading to loss of vision and retinal re-detachment, and no validated drugs are available for the prevention or treatment of PVR.^1^^,^^5^^–^^7^ Antivascular endothelial growth factor (anti-VEGF) therapy is primarily used to treat nAMD; however, inflammation and fibrosis cannot be controlled through this therapeutic approach.^8^^–^^12^ Therefore, the development of new treatment strategies is required to effectively manage AMD and PVR fibrosis.

The epithelial-to-mesenchymal transition (EMT) of the retinal pigment epithelium (RPE) involved in the pathogenesis of intraocular fibrosis associated with AMD and PVR is characterized by the fibroblastic phenotype and enhanced proliferative and migratory abilities of RPE cells. Additionally, these cells acquire the ability to synthesize extracellular matrix (ECM), a critical process that facilitates the formation of fibrotic membranes.^3^^,^^4^^,^^13^^,^^14^ Several animal-based studies have revealed the suppressed progression of fibrosis in PVR and nAMD through inhibited EMT in the RPE.^15^^–^^18^ Therefore, targeting EMT in RPE cells is considered a promising therapeutic strategy for the inhibition of fibrosis progression.

Rho-kinase (ROCK), a downstream effector of Rho, plays vital roles in cellular processes such as adhesion, migration, proliferation, and apoptosis.^19^ Elevated activity of the ROCK pathway has been identified as a contributing factor in the development of several eye diseases, including PVR and AMD.^20^^,^^21^ Moreover, ROCK inhibitors can suppress PVR formation and progression^22^ and reduce the volume of fibrosis in the choroidal neovascularization (CNV) model.^20^ Moreover, a recent study demonstrated that ROCK signaling contributes to subretinal fibrosis through EMT in RPE cells, and ripasudil (Ripa), a highly potent ROCK inhibitor, suppresses subretinal fibrosis in a mouse model of laser-induced CNV by inhibiting EMT.^23^ Therefore, Ripa can be regarded as a promising therapeutic agent for the management of intraocular fibrosis through EMT inhibition.

However, the pharmacokinetic profile of Ripa limits its therapeutic potential. Administering treatments topically presents challenges in achieving effective concentrations in the posterior segment of the eye.^24^ For intravitreal injections, despite high cell permeability, small-molecule drugs have short vitreal half-lives (1–27 hours in rabbits).^25^ Therefore, a formulation equipped with a sustained-release delivery system is required to minimize the need for frequent dosing.^26^

Nanotechnology, a recently emerging transformative approach in the field of medicine, supports effective strategies for drug delivery to the posterior chamber of the eye and enhances the in vivo stability of low-molecular-weight compounds.^27^^,^^28^ Liposome encapsulation, a leading technology in this field, can maximize therapeutic outcomes by enhancing drug stability, controlling release rates, and improving cellular uptake.^29^^–^^35^

In this study, we aimed to investigate the potential of liposome-encapsulated ripasudil (Lipo-Ripa) in alleviating intraocular fibrosis, which can pave the way to a new era in the therapeutic management of PVR and nAMD.

## Methods

### Preparation of Lipo-Ripa

Empty polyethylene glycol (PEG) liposomes were composed of HSPC/CHOL/mPEG2000-DSPE (9.58/3.19/3.19, weight ratio; and 56.3/38.4/5.3, mol%, respectively). Hydrogenated soy phosphatidylcholine (HSPC) was purchased from Lipoid GMBH (Ludwigshafen, Germany). A portion of 1,2-distearoyl-sn-glycero-3-phosphoethanolamine-n-[methoxy (polyethylene glycol)-2000] (mPEG2000-DSPE) was donated by NOF Corp., and the rest of the components were purchased from Genzyme Pharmaceuticals (Liestal, Switzerland). Cholesterol (CHOL) was purchased from Nipponseika Co., Ltd. (Osaka, Japan). All the lipids were used without further purification. The highest available grade of all other reagents was used. Ripasudil powder was obtained from Kowa Company, Ltd, Japan.

The lipids, dissolved in ethanol, were added to 250 mM ammonium sulfate solution at a ratio of 1:20 (vol/vol; lipid: ammonium sulfate), followed by mixing and stirring. The liposomes obtained were sorted and extruded through polycarbonate membrane filters (Whatman Inc., Florham Park, NJ, USA) with pore sizes of 400 and 200 nm, and the external buffer was exchanged by ultrafiltration using a polyethersulfone membrane (Millipore Corp., Burlington, MA, USA). The obtained liposomal suspension was ultracentrifuged at 452,000 × g using a Himac CP80NX (Hitachi Koki, Tokyo, Japan) for 60 minutes. The precipitate was adequately dispersed in the diluent to prepare the stock liposome suspension. Ripasudil was encapsulated in liposomes using a remote-loading method. The ripasudil stock solution (4 mg/mL) was mixed with the stock liposome at a ratio of 1:1 (v/v) and diluted using the diluent to finally obtain 2 mg/mL ripasudil suspension, which was subsequently incubated in a water bath maintained at 60°C for 5 minutes and occasionally shaken for remote loading.^36^ Subsequently, the solution was cooled to 25°C and used as the ripasudil liposome in further analyses. The mean diameters and Z-potential of the prepared liposomes were determined using a Zetasizer Nano-ZS (Malvern Instruments Ltd., Worcestershire, UK).

### Liquid Chromatography-Tandem Mass Spectrometry 

The ripasudil concentration was measured using liquid chromatography-tandem mass spectrometry (LC-MS/MS). The protein precipitation solvent (100% acetonitrile) was added to the samples at a ratio of 50:50 (v/v). Plasma was added to the protein precipitation solvent and separated by centrifugation (12,000 revolutions per minute [rpm] for 20 minutes). The supernatant was analyzed using LC-MS/MS. The drugs were resolved using an AQUITY UPLC HSS PFP column (100 mm × 2.1 mm, 1.8 µm, p/n186003461; Waters, Milford, MA, USA) on an AQUITY UPLC H-Class XHCLQT0100 system (Waters) equipped with a vacuum degasser, binary pump, thermostatically controlled column compartment, thermostatically controlled autosampler, and diode array detector. Mobile-phase A contained 0.1% formic acid/40% water/60% acetonitrile and mobile-phase B contained 0.1% formic acid/90% 2-propanol/10% acetonitrile. A linear gradient was generated at 0.5 mL/min: 0 minutes, 60% A and 40% B; 0 to 4 minutes, 50% A and 50% B; and 4 to 7 minutes, 60% A and 40% B in accordance with the application note provided by the manufacturer (Waters). The injection volume was 10 µL. The temperature in the column autosampler compartment was maintained at 40°C and 10°C. An AQUITY TQ mass spectrometer controlled by Masslynx software (Waters) was used in the selected reaction-monitoring mode. The multiple reaction monitor was set at mass-to-charge ratios (m/z) of 324–99 m/z (cone voltage = 20 and collision voltage = 30) for ripasudil and 369.2–95 m/z (cone voltage = 166 and collision voltage = 40) for cholesterol (Nipponseika).

### Cryo-Electron Tomography

Cryo-electron tomography of frozen hydrated liposome specimens was conducted using Holey carbon grids (Quantifoil R1.2/1.3 Cu 200 grids; Quantifoil Micro Tools GmbH, Germany). Before use, the grids were glow-discharged for 1 minute using a GloQube Plus hydrophilic treatment device (Quorum) at a discharge current of 40 milliampere (mA). Liposome suspension (3 µL) was added to the grids, and rapid freezing was performed using a VItrobot Mark IV device (Thermo Scientific, Waltham, MA, USA) at 4°C and 100% humidity.

Cryo-electron tomographic data were collected using a CRYO ARM 200 (JEM-Z200FSC; JEOL, Tokyo, Japan) equipped with a cold field-emission electron gun (cold FEG), an omega energy filter (zero-energy-loss mode, slit width 20 eV), and a Gatan K3 direct detector (AMETEK Gatan, USA) at an accelerating voltage of 200 kV. Tilt series images were acquired automatically at a nominal magnification of 25,000 × using SerialEM,^37^ covering an angular range of ± 60 degrees. The acquisition used a dose-symmetric scheme, with 3 degrees tilt increments. The target defocus was set to −3 µm. The K3 camera was operated in the counting mode (CDS mode) at a dose rate of 8.5 electrons/pixel/s. Each tilted image was fractionated into 10 frames and aligned during the SerialEM recording procedure. The total electron dose of the tilt series was approximately 100 electrons/A2.

Alignment of the tilt series images and reconstruction of the tomograms were performed using patch tracking and the weighted back-projection method was implemented in IMOD software.^38^ Three-dimensional segmentation of liposomes and ripasudil was performed using a convolutional neural network (CNN)-based semi-automated annotation protocol^39^ in EMAN2.^40^ A chimera was used to visualize the tomogram and segmented 3D model.^41^

### In Vitro Drug-Release Assay

To evaluate the drug release of Ripa-loaded liposomes in different pH environments, Lipo-Ripa was mixed in pH 6.0 or 7.4 phosphate buffers and incubated at 37°C for 1 to 2 hours. The samples were then centrifuged at 60,000 rpm for 60 minutes. The supernatant was separated to the new tubes and the pellet (Lipo-Ripa) was suspended in methanol. The Ripa concentration was measured using LC-MS/MS.

### Cells

Primary RPE cells were obtained from Lonza (Walkersville, MD, USA) and cultured following previously reported protocols.^15^^,^^42^ The previously described comprehensive methodology for establishing highly polarized and functional RPE monolayers was adopted.^43^^,^^44^ In brief, the cells were cultured for 1 week at 37°C in a controlled environment with 5% CO_2_ using RtEGM growth medium (Lonza Walkersville Inc.) supplemented with 2% fetal bovine serum (FBS). Subsequently, approximately 1.0 × 10^5^ RPE cells were seeded on fibronectin-coated Transwell inserts (10.5 mm internal diameter, 0.4 µm pore size, Transwell; BD Falcon, USA) and incubated for 3 weeks or longer (Supplementary Fig. S1a). The culture medium was replenished every 3 days. The transepithelial resistance (TER) of the RPE cell monolayers was measured using an Epithelial Voltohmmeter 2 (EVOM2; World Precision Instrument, Sarasota, FL, USA) following previously reported protocols.^43^^,^^45^ After achieving a minimum TER of 400 Ω·cm², RPE cells of the same batch were concurrently used in all the experiments.

### Drug Screening

The ophthalmic medications cyclosporine (CSA), latanoprost (LAN), timolol (TIM), tranilast (TRAN), and Ripa were used at appropriate concentrations to treat RPE cells, as previously described.^46^^–^^49^ RPE cells were cultured using Dulbecco’s Modified Eagle's Medium (DMEM) supplemented with 2 mM L-glutamine, 100 U/mL penicillin, 100 µg/mL streptomycin, and 10% FBS. Subsequently, the cells were subjected to overnight starvation in serum-free media, which was then replaced with DMEM containing 3% FBS. The cells were pretreated using CSA (31.6 µM), LAN (10 µg/mL), TIM (6 µM), TRAN (300 µM), and Ripa (30 µM) for 6 hours, followed by stimulation with recombinant transforming growth factor (TGF)-β2 (10 ng/mL; Sigma-Aldrich, St. Louis, MO, USA) for 48 hours. The cells were then harvested for RNA extraction.

### RNA Isolation and Reverse Transcription-Quantitative Polymerase Chain Reaction

Total RNA was extracted from the treated cells using the RNeasy kit (QIAGEN, Valencia, CA, USA) following the protocol provided by the manufacturer. RT-PCR was performed as reported in a previous study.^50^ Glyceraldehyde-3-phosphate dehydrogenase (*GAPDH*) was considered a reference gene for the normalization of gene expression levels in terms of the fold-change relative to the standard controls. The following primer pairs were used in the process:

E-cadherin, 5′-ATTTTTCCCTCGACACCCGAT-3′ and 5′-TCCCAGGCGTAGACCAAGA-3′;
α-SMA, 5′-TCTGTAAGGCCGGCTTTGC-3′ and 5′-TGTCCCATTCCCACCATCA-3′; and
GAPDH, 5′-ACAGTCGCCGCATCTTCTT-3′ and 5′-CT TGATTTTGGAGGGATCTCGC-3′.

### Cellular Lipo-Ripa Uptake Measurements

RPE cells were cultured as previously described and subsequently plated into six-well plates. After attaining 80% confluence, the cells were subjected to overnight starvation using DMEM supplemented with 3% FBS. Afterward, the cells were exposed to media either with or without TGF-β2 at a concentration of 10 ng/mL for 48 hours. Subsequently, they were incubated in a medium infused with 30 µM Lipo-Ripa. At 6 hours post-incubation, the cells were washed with Balanced Salt Solution (BSS), trypsinized, and subsequently centrifuged.

The homogenate from collected cells in distilled water were prepared using a Dounce homogenizer and centrifuged at 12,000 rpm for 10 minutes. The intracellular Ripa in the supernatant was measured using LC-MS/MS.

### Permeability Assay

To assess the permeability of Ripa and Lipo-Ripa across the RPE layer, both drugs, diluted to 30 µM using BSS (Alcon, Japan), were separately introduced into the upper inserts. Moreover, BSS was added to the lower chamber. The medium was collected from the lower chamber at different time intervals (1, 3, 6, 12, 24, and 48 hours) and submitted to Kowa Company for LC-MS/MS analysis to explore the concentration of Ripa in the collected samples pretreated with methanol. The penetration rate of Ripa was calculated using the following formula:

$$\begin{array}{ccc} \mathrm{Penetration}\;\mathrm{rate}\;(\%)\; & = & \;(\mathrm{Amount}\;\mathrm{of}\;\mathrm{Ripa}\;\mathrm{in}\;\mathrm{the}\;\mathrm{lower}\\ & & \;\mathrm{chamber})/(\mathrm{initial}\;\mathrm{amount}\;\mathrm{of}\;\mathrm{total}\;\mathrm{Ripa}). \end{array}$$

The permeability coefficient was determined using the following formula:

$$\begin{array}{ccc} & & \;\mathrm{Permeability}\;\mathrm{coefficient}\;\left(\mathrm{cm}/h\right)\;\\ & & \;\;=(\mathrm{Velocity}\;\mathrm{of}\;\mathrm{Ripa}\;\mathrm{passage}\;\mathrm{through}\;\mathrm{the}\\ & & \;\mathrm{RPE}\;\mathrm{monolayers}\;(\mathrm{mol}/h))/\\ & & \;(\mathrm{Cell}\;\mathrm{culture}\;\mathrm{area},\;0.9{\;\mathrm{cm}}^{2})\;\\ & & \;\times \;(\mathrm{Initial}\;\mathrm{Ripa}\;\mathrm{concentration}\;\mathrm{in}\;\mathrm{the}\;\mathrm{medium}\\ & & \;\mathrm{present}\mathrm{in}\;\mathrm{the}\;\mathrm{upper}\;\mathrm{chamber}\;(\mathrm{mol}/{\mathrm{cm}}^{3})). \end{array}$$

### Animals

All the animal experiments were approved by the appropriate institutional committee. For in vivo studies, we used 4 mice per group for each experiment (32 in total); 3 rabbits per group for PVR stage tracking, 2 rabbits per group for ELISA, 2 rabbits for the toxicity study, and 12 rabbits for drug concentration (31 in total). For in vitro experiments, each condition was tested in triplicate with at least three independent biological replicates. Wild-type (WT) C57BL/6J male mice (Charles River Laboratories Japan, Yokohama, Japan) aged between 8 and 10 weeks were used in all the studies to eliminate sex-related biases in the size of the laser-induced CNV lesions.^51^ Dutch rabbits weighing 2 to 2.5 kg were used to establish the PVR models. All the animal experiments were conducted following the guidelines of the Association for Research in Vision and Ophthalmology Statement for the Use of Animals in Ophthalmic and Vision Research.

### Laser-Induced CNV Model

CNV models were generated following a previously reported protocol.^52^ In summary, on day 0, laser photocoagulation (532 nm, 100 mW, 50 ms, and 75 µm) of the fundus was performed in WT mice (male, age 6–8 weeks) using a coverslip as a contact lens. The presence of a subretinal bubble during laser application indicated the rupture of the Bruch’s membrane. Four laser spots were created in each eye for immunofluorescence staining assay and 20 laser spots were generated per eye for further protein extraction required to assess ROCK activity. Mice that exhibited burns or bleeding during the procedure were excluded. Considering a previous report of the reduction in CNV by day 14 after laser photocoagulation,^16^ single intravitreal injections of 1 µL of BSS (control group), Ripa (30 µM), and Lipo-Ripa (30 µM) were administered to each respective group on day 14 post-laser injury. On post-laser injury day 28, the mice were euthanized, and their eyeballs were collected; subsequently, the choroidal flat mounts were prepared and protein was extracted.

### Rabbit PVR Model

PVR was induced using Dutch rabbits (male, weighing 2.0–2.5 kg), following a previously reported protocol.^53^^,^^54^ After 1 week of acclimatization, gas vitrectomies were performed by injecting 0.1 mL of perfluoropropane (C3F8; Alcon) into the vitreous cavity 3 mm posterior to the corneal limbus. One week after this procedure, 0.1 mL DMEM containing 2.5 × 10^5^ RPE cells and 40 ng recombinant human PDGF (R&D, 220-BB) were administered through intravitreal injections in both eyes of each rabbit. Ophthalmoscopy (Retcam 3 ophthalmic imaging system; Natus, USA) was performed to record the progression of PVR, categorized based on Fastenberg's classification.^53^ For the efficacy study, 3 days after injecting RPE cells, 0.1 mL BSS (control), Ripa (2 mM), and Lipo-Ripa (2 mM) were injected into the vitreous cavity in each experimental group. Enzyme-linked immunosorbent assay (ELISA) was performed using vitreous samples collected from rabbits euthanized 14 days after drug administration, whereas clinical ophthalmoscopic examinations were conducted up to 28 days after drug injections.

For the pharmacokinetic study, each rabbit was administered 0.1 mL of Ripa (2 mM) and 0.1 mL of Lipo-Ripa (2 mM) through intravitreal injection into the right and left eyes, respectively, 1 week after RPE cell injections. The rabbits were euthanized at 6, 24, 72, and 120 hours after these injections, and their eyeballs were enucleated to collect vitreous and retinal samples. After the retinal samples were homogenized and pretreated with methanol, the concentrations of Ripa in vitreous and retinal samples were detected by Kowa Company (lower limit of quantification = 2 nM) using LC-MS/MS. For quantification of drug distribution in ocular tissues, the rabbit vitreous volume was assumed to be 1.5 mL, the retinal tissue weight was estimated at 45 mg, as reported in previous pharmacokinetic studies.^55^^,^^56^

### Measurement of CNV and Choroidal Fibrosis Volumes

The volumes of the CNV and subretinal fibrous tissue were measured using choroidal flat mounts prepared 28 days after laser photocoagulation. Mouse eyecups were fixed in 4% paraformaldehyde and permeabilized by incubating in 1% Triton X-100 (ICN Biomedicals, Irvine, CA, USA) for 2 hours. After the removal of the anterior segment and neural retina, fluorescein-labeled isolectin-B4 (Vector Laboratories, Burlingame, CA, USA) and collagen type I antibody (Rockland Immunochemicals Inc., Limerick, PA, USA) were added to the eyecups, followed by overnight incubation at 4°C for the detection of CNV and subretinal fibrous tissue. A goat anti-rabbit IgG secondary antibody with Alexa Fluor 647 conjugate (Life Technologies, Grand Island, NY, USA) was added as the secondary antibody against collagen type I. The samples were coverslipped and examined using a confocal microscope (Nikon). Fluorescence volume was quantified using image stacks of optical slices generated within the lesions, following a previously described method.^57^

### ELISA

Periostin concentrations were measured using a Periostin ELISA Kit (Shino-test, Co., Tokyo, Japan) following the previously described protocol.^58^^,^^59^

### ROCK Activity Assay

The relative RhoA/ ROCK activation was evaluated in the CNV models using a commercially available ROCK activity EIA kit (Cell Biolabs, Inc., San Diego, CA, USA) according to the instructions provided by the manufacturer. For this assay, on day 14 post-laser irradiation, single doses of each drug were administered through intravitreal injection. Proteins were then isolated from the sonicated RPE/choroid complexes with 20 laser spots using a tissue protein extraction reagent with a protease inhibitor (Thermo Scientific) 28 days post-laser irradiation. Subsequently, the protein samples were incubated on 96-well plates coated with myosin phosphatase target subunit 1 (MYPT1), along with dithiothreitol and ATP-containing kinase buffer, for 1 hour at 30°C. Anti-phospho-MYPT1 (Thr969) was then added, followed by washing and incubation with a horseradish peroxidase-conjugated secondary antibody. Finally, the plate was developed using a substrate solution and the absorbance was measured at a primary wavelength of 450 nm using a spectrophotometer.

### Liposomes Labeled With Fluorescein DHPE

Fluorescein-DHPE (N-(fluorescein-5-thiocarbamoyl)-1,2-dihexadecanoyl-sn-glycero-3-phosphoethanolamine, triethylammonium salt; F-362, Invitrogen) was used to label the liposomes. To track the liposomes, fluorescein-DHPE, and liposomes labeled with fluorescein-DHPE were added to polarized RPE cells or injected into the eyes of the mouse CNV models.

### Immunofluorescence Staining in the Polarized RPE

Immunofluorescent staining for ZO-1 was performed separately using polarized RPE cells, as previously described.^44^ RPE cells were first permeabilized by incubating them in phosphate-buffered saline containing 0.2% Triton X for 30 minutes followed by fixation in ice-cold methanol (4°C) for 15 minutes. The specimens were blocked with 5% bovine serum albumin and incubated with 1:100 ZO-1 primary antibody (61-7300; Thermo Fisher Scientific). Incubation with rabbit secondary antibodies (Alexa Fluor; Molecular Probes) was conducted for 30 minutes in the dark at room temperature. The nuclei were counterstained using Hoechst33342 (H3570; Invitrogen). All the specimens were visualized under a fluorescence microscope (BZ-X710; Keyence, Osaka, Japan).

### Ocular Toxicity Assessment in Rabbits

To evaluate the ocular safety of Lipo-Ripa, we conducted additional toxicity assessments in rabbit eyes. Lipo-Ripa was administered via intravitreal injection into the left eye of each rabbit at a dose consistent with that used in the efficacy experiments (0.1 mL and 2 mM), whereas the right eye injected with 0.1 BSS served as a control. After injection, intraocular pressure (IOP) was measured in both eyes every 7 days for a total of 4 weeks using a Icare Pro TA03 tonometer (Tokyo). Following the 4-week observation period, the rabbits were euthanized, and their eyes were enucleated for further analysis. Fundus imaging was performed using the RetCam system to assess the retinal condition, and paraffin-embedded ocular sections were prepared for histological evaluation. Hematoxylin and eosin (H&E) staining was performed to detect any signs of intraocular inflammation, retinal damage, or other pathological changes.

### Statistical Analysis

All results are expressed as means ± SEM or SD. The statistical significance of the differences between data corresponding to different groups was analyzed using Tukey's test or a two-tailed *t*-test. Dunnett's *t*-test was used to compare the data with that of the control group. Differences were considered statistically significant at *P* < 0.05. Statistical analyses were performed using GraphPad Prism 9 (La Jolla, CA, USA) software.

## Results

### Drug Screening

To identify effective drugs for suppressing EMT in RPE cells, we examined the altered expression of E-cadherin (epithelial marker) and α-smooth muscle actin (α-SMA, mesenchymal marker) in RPE cells treated with CSA, LAN, TIM, TRAN, and Ripa. These compounds are specifically used in the treatment of ocular fibrosis diseases. TGF-β2, the most prevalent TGF-β isoform in the posterior segment of the eye, plays a critical role in triggering EMT in RPE cells.^60^ Exposure to TGF-β2 (10 ng/mL) for 48 hours markedly diminished the expression of E-cadherin and upregulated α-SMA at the mRNA level.^15^

**Figure 1.**
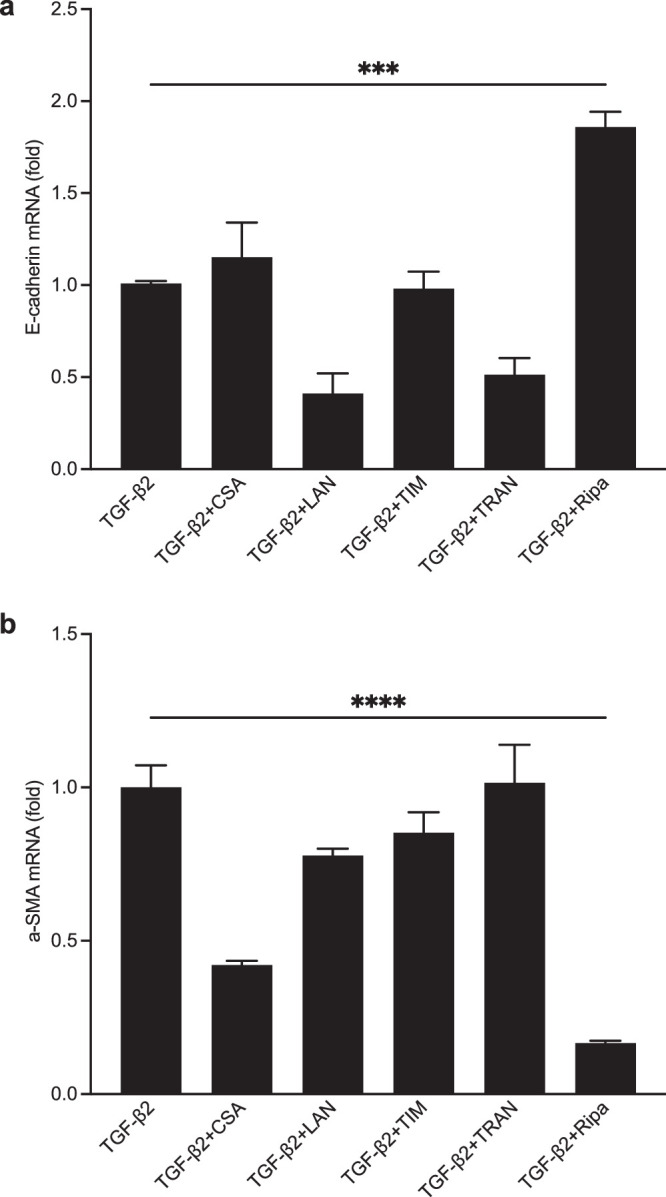
Screening of drugs for efficient inhibition of EMT of RPE cells. RPE cells were pretreated with either blank culture medium (control) or medium containing cyclosporine (CSA), latanoprost (LAN), timolol (TIM), Tranilast (TRAN), or ripasudil (Ripa) for 6 hours. Next, they were stimulated with 10 ng/mL transforming growth factor (TGF)-β2 for 48 hours. (**a**, **b**) The mRNA expression of E-cadherin and a-SMA, expressed as relative fold change relative to the control group (normalized using GAPDH). Data are presented as means ± SEM, *n* = 4, Dunnett’s test (*** *P* < 0.001, **** *P* < 0.0001). EMT, epithelial-to-mesenchymal transition; RPE, retinal pigment epithelial cells.

Our results indicated that Ripa treatment considerably increased E-cadherin and decreased α-SMA at the mRNA level simultaneously, whereas other test drugs did not show equivalent efficacy (Figs. 1a, 1b). Therefore, the TGF-β2-induced decrease in E-cadherin and increase in α-SMA could be inhibited using Ripa, which is consistent with a previous report.

### Physicochemical Properties of Lipo-Ripa

**Figure 2.**
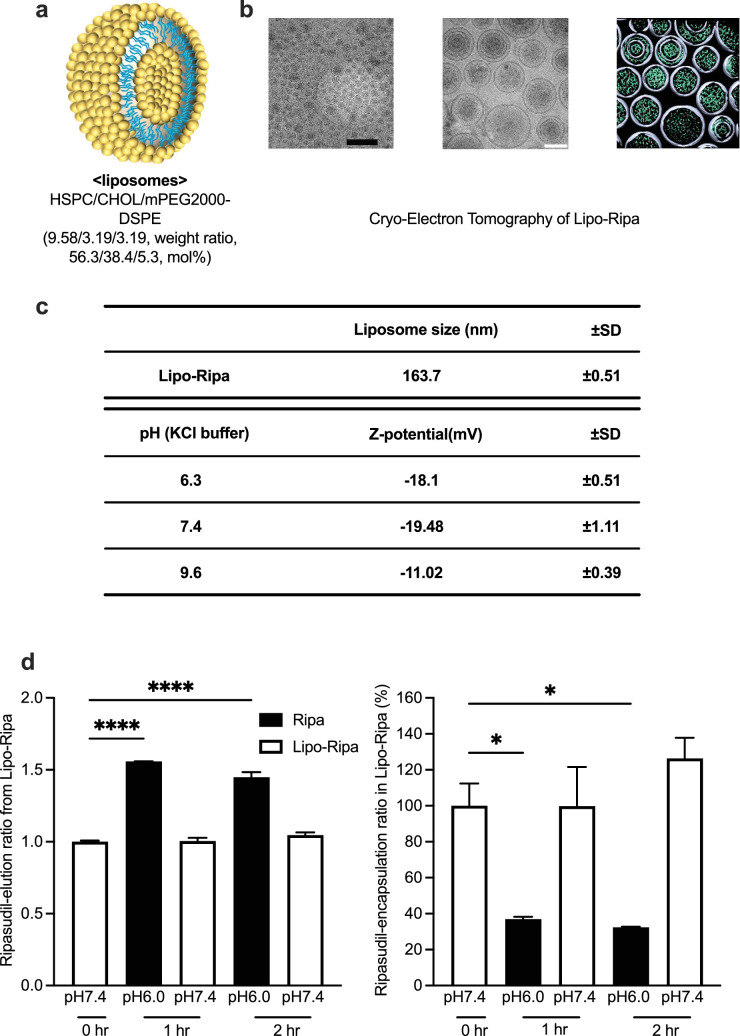
Physicochemical properties of liposome-encapsulated Ripasudil (Lipo-Ripa). (**a**) Schematic drawing of liposomes. (**b**) Cryo-electron microscopic images (*left, middle*) of Lipo-Ripa recorded using the JEOL CRYO ARM 200 and segmented tomogram (*right*; surface of liposome: *light gray*, a high electron density region: *light green*). Scale bar = 500 nm (*black*) and 100 nm (*white*). (**c**) The particle size and Z-potential of liposomes are shown in the supplementary tables. (**d**) Elution ratio (*left*) and encapsulation ratio (*right*) expressed as relative fold change, compared with the control group (pH 7.4, 0 hours). Data are presented as means ± SEM, *n* = 3, Dunnett’s test (* *P* < 0.05, **** *P* < 0.0001).

The schematic diagram and composition of the liposomes used in this experiment are depicted in Figure 2a. Cryo-electron microscopy images and segmented tomograms (Fig. 2b) indicate that most of the Lipo-Ripa were spherical and unilamellar, and approximately 100 to 200 nm. These data suggest that Ripa is encapsulated in the lipid membranes. The physicochemical properties of Lipo-Ripa were evaluated in terms of particle size, Z-potential, and morphology. The particle size of Lipo-Ripa was 163.7 ± 0.51 nm. Lipo-Ripa was identified as an anionic liposome using Z-potential analysis (Fig. 2c). Lipo-Ripa demonstrates stability in environments with a pH of 7.4, with < 1% of the Ripa content being detected as the unencapsulated drug in the formulation, as determined by LC-MS/MS analysis. However, after incubation in an environment with a pH of 6.0 for 1 to 2 hours, the elution rate of Ripa from the Lipo-Ripa formulation markedly increased (*P* < 0.0001), and the encapsulation efficiency markedly decreased (*P* < 0.05; Fig. 2d).

### Pharmacokinetic Study In Vivo and In Vitro

We established polarized RPE monolayers using Transwell culture systems to assess the permeability of Ripa and Lipo-Ripa through polarized RPE cells, as detailed in the Materials and Methods section (see Supplementary Fig. S1a). Notably, remarkable pigmentation was evident after 21 days of incubation (see Supplementary Fig. S1b), and the TER remained stable at ≥ 400 Ω·cm² for up to 2 months following 28 days of incubation (see Supplementary Fig. S1c).

In the permeation study on the polarized RPE, immunofluorescence analysis revealed no discernible alterations in the intercellular junctions of cells labeled for ZO-1 (see Supplementary Fig. S1d).

**Figure 3.**
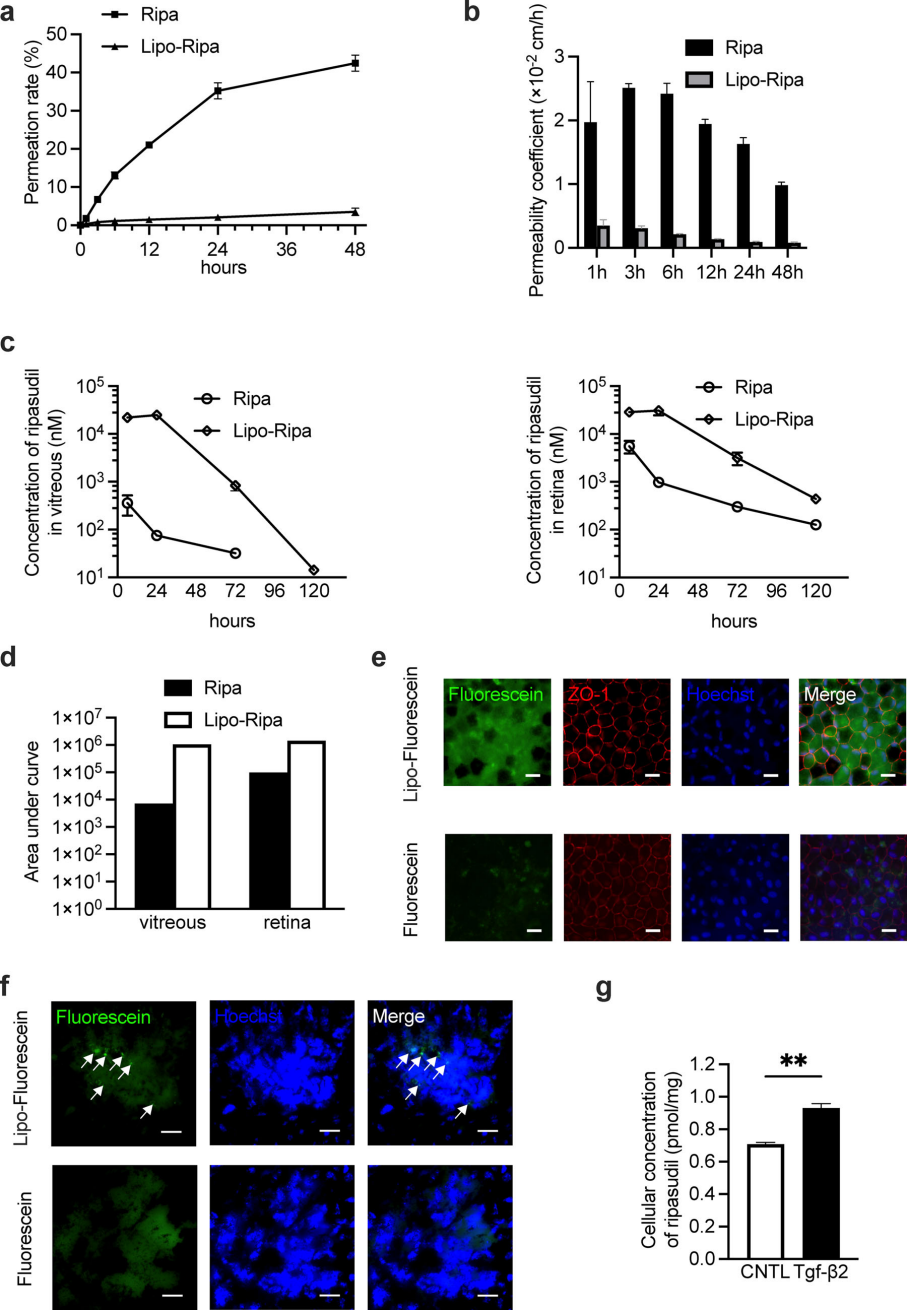
In vivo and in vitro pharmacokinetic analysis of Ripa and Lipo-Ripa. (**a**) Permeability of Ripa and Lipo-Ripa through the retinal pigment epithelium (RPE) layer. At all time points, the penetration ratio of Ripa is significantly greater than that of Lipo-Ripa. After 48 hours, approximately 40% of Ripa permeated the RPE monolayer, whereas < 4% of Lipo-Ripa traversed the RPE monolayer. Data are presented as mean ± SEM, *n* = 4, *t*-test (* *P* < 0.05, **** *P* < 0.0001). (**b**) Permeability coefficients of Ripa and Lipo-Ripa. The calculation formula is described in the Materials and Methods section. (**c**) Changes in Ripa concentration over time in the vitreous and retina after intravitreal injection of 0.1 mL of either 2 mM Ripa or 2 mM Lipo-Ripa. The minimum concentration detection limit of Ripa is 2 nM. Data are presented as mean ± SEM, *n* = 3. (**d**) The relative AUC of the Lipo-Ripa group is approximately 146.46, compared with that of the Ripa group, whereas the relative AUC of the Lipo-Ripa group, compared with that of the Ripa in the retina is approximately 14.6. (**e**) Representative pictures indicating fluorescein signal within the polarized RPE system. Fluorescein-DHPE and Fluorescein-DHPE-labeled liposomes (Lipo-Fluorescein) were added to the culture medium and introduced into the upper chamber of the Transwell system. After 48 hours of incubation, the Transwell membranes were collected and stained with DAPI. Scale bar = 20 µm. (**f**) Representative pictures showing the lesion in choroidal flat mounts 2 days after injecting Fluorescein-DHPE and Lipo-Fluorescein at 14 days post-laser induction. Fluorescein signals seen in the lesion are indicated by *arrows*. Cell nuclei were stained using Hoechst. Scale bar = 20 µm. (**g**) Cellular concentration of Ripa in RPE cells treated with or without TGF-β2. Data are presented as mean ± SEM, *n* = 3, *t*-test (**P < 0.01). AUC, area under the curve.

The Ripa group exhibited markedly greater permeation than the Lipo-Ripa group at all the measured intervals. Specifically, 40% of Ripa contrasted with < 4% of Lipo-Ripa that permeated the monolayer after 48 hours (Fig. 3a). The permeability coefficients of Ripa far exceeded those of Lipo-Ripa (Fig. 3b). In addition, after 48 hours, quantitative analysis showed that the average proportion of free ripasudil in the upper chamber was 19.3% in the Lipo-Ripa group, suggesting that most of the drug remained encapsulated within liposomes. In contrast, in the lower chamber, ripasudil was detected predominantly in the free form, with an average free ratio of 97.3% in the Lipo-Ripa group, implying that unencapsulated ripasudil was primarily responsible for permeation across the RPE monolayer (Supplementary Fig. S2a).

Pharmacokinetic assessments in vitreous and retinal tissues revealed substantially higher concentrations and areas under the curve (AUCs) for the Lipo-Ripa group, compared with the Ripa group. This indicated a pronounced retention and extended presence of the drug within the eye (Figs. 3c, 3d).

Immunofluorescence revealed that RPE cells internalized more fluorescein-DHPE when delivered with liposomes, indicating that liposomal encapsulation enhances cellular uptake of the dye (Fig. 3e). We conducted experiments in the laser-induced CNV model. Additionally, in a CNV model, liposomes labeled with fluorescein-DHPE showed remarkable aggregation at lesion sites, contrasting with the lack of signal from the dye alone (Fig. 3f). This suggested that liposomal delivery effectively targeted the affected areas. Subsequently, we found that after 48 hours of TGF-β2 induction, the uptake of Lipo-Ripa by the RPE cells increased, which was statistically significant (*P* < 0.01; Fig. 3g).

To further characterize the release kinetics of Lipo-Ripa, we analyzed the proportion of free ripasudil in the vitreous over time, the free drug ratio was 69.4% at 24 hours, slightly decreased to 66.7% at 72 hours, and then increased to 92.45% by 120 hours, indicating a gradual release of ripasudil from the liposomal formulation (see Supplementary Fig. S2b).

We also evaluated the partitioning of ripasudil between the vitreous and retina. Results demonstrated that Lipo-Ripa exhibited a time-dependent increase in the retina/vitreous ratio, rising from 4.2% at 6 hours to 16.4% at 72 hours, and eventually reaching 100% at 120 hours as drug levels equilibrated. In contrast, Ripa exhibited faster retinal penetration, with an initial retina/vitreous ratio of 26.4% at 6 hours, increasing to 39.5% at 24 hours, and remaining at 29.7% by 72 hours (see Supplementary Fig. S2c). These data clearly demonstrate that Lipo-Ripa functions as a sustained-release intravitreal formulation, providing a prolonged reservoir in the vitreous that enables gradual delivery to the retina.

### Efficacy of Lipo-Ripa in Subretinal Fibrosis

**Figure 4.**
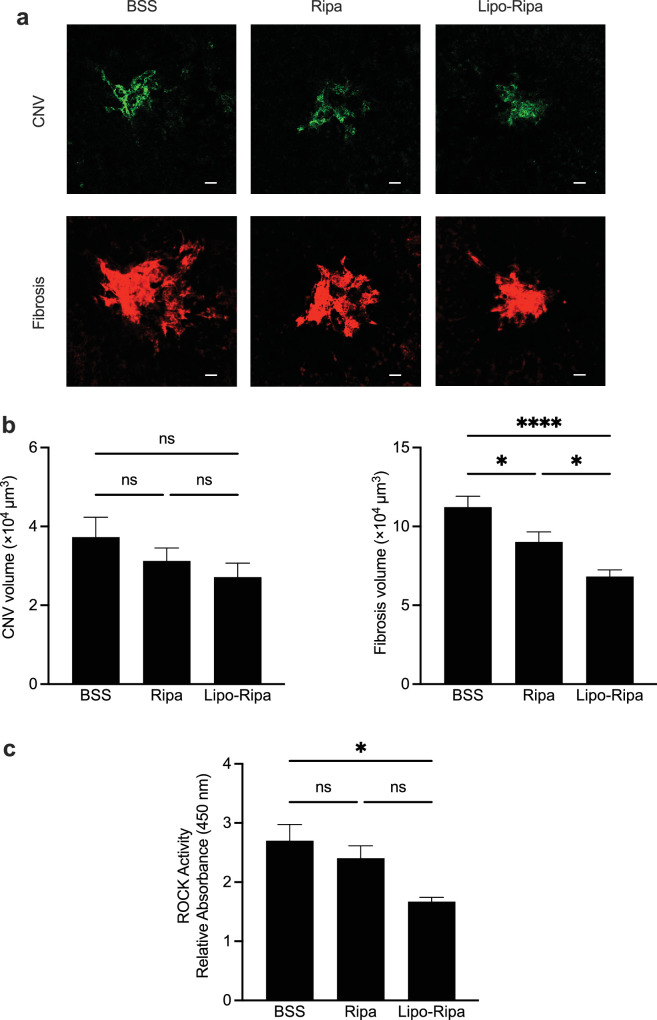
Inhibitory effect of Lipo-Ripa on subretinal fibrosis in laser-induced choroidal neovascularization (CNV) model. (**a**) Representative pictures of CNV (stained by isolectin B4, *green*) and fibrosis (stained by collagen type I, *red*) at day 28 after laser induction in each group. Scale bar = 20 µm. (**b**) The mean volume of CNV and fibrosis 28 days after laser induction in the eyes in the BSS-, Ripa-, and Lipo-Ripa-treated groups. Data are presented as means ± SEM, *n* = 15, Tukey's test (* *P* < 0.05, **** *P* < 0.0001). (**c**) ROCK activity in the protein extracted from choroidal tissue 28 days after treatment with drugs. Data are presented as means ± SEM, *n* = 4, Tukey's test (* *P* < 0.05). ns, not significant.

The pivotal characteristics of nAMD, CNV, and fibrosis in the mouse laser CNV model were thoroughly investigated. During the critical transition phase from CNV to fibrosis, drugs were administered on day 14 post-laser treatment, potentially enhancing the efficacy of drugs against fibrotic pathways and contributing to the development of therapies for advanced nAMD. Confocal microscopic analysis was conducted to quantify the volumes of CNV (labeled with Abs against isolectin B4) and SRF (labeled with collagen type I) using RPE/choroid flat mounts 28 days after laser injury. The results revealed no statistically significant differences in the CNV volume among the control, Ripa-treated, and Lipo-Ripa-treated groups. However, analysis of fibrosis revealed markedly reduced volumes of type 1 collagen-positive cells in both the Ripa and Lipo-Ripa groups compared to that in the control group. Furthermore, Lipo-Ripa exhibited enhanced effectiveness, compared with Ripa treatment (Figs. 4a, 4b). Notably, Lipo-Ripa more effectively alleviated subretinal fibrosis, compared with an equivalent dose of Ripa. ROCK activity showed a more decreasing trend in both the Ripa and Lipo-Ripa treatment groups compared with the control group. However, a statistically significant difference was only observed in the Lipo-Ripa group (*P* < 0.05; Fig. 4c), which indicated the enhanced inhibitory effect of Lipo-Ripa on ROCK, compared with that of Ripa treatment.

### Efficacy of Lipo-Ripa in the Experimental Model of PVR

**Figure 5.**
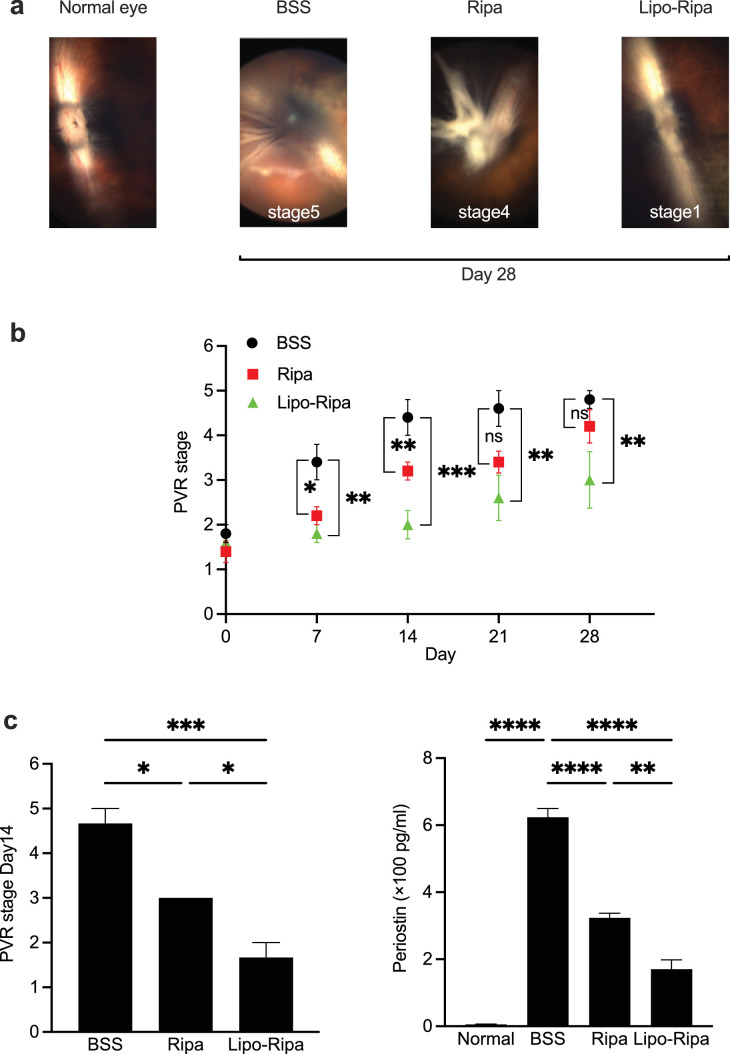
Efficacy of Lipo-Ripa in experimental model of proliferative vitreoretinopathy (PVR). (**a**) Photographs showing the ocular fundus of the PVR eyes after treatment with BSS, Ripa (2 mM), and Lipo-Ripa (2 mM). (**b**) Progression of PVR stages in the rabbit model injected with BSS, Ripa (2 mM), and Lipo-Ripa (2 mM) on day 0. Data are presented as mean ± SEM. *n* = 5, Dunnett's test (* *P* < 0.05, ** *P* < 0.01, *** *P* < 0.001). (**c**) PVR stage and concentrations of periostin, a fibrotic factor, in the vitreal samples analyzed in each group using ELISA 14 days after injection. Data are presented as mean ± SEM, *n* = 3, Tukey's test (* *P* < 0.05, ** *P* < 0.01, *** *P* < 0.001, **** *P* < 0.0001). BSS, balanced salt solution.

We compared various stages of PVR in rabbit eyes among BSS (control), Ripa-treated, and Lipo-Ripa-treated groups through fundus image analysis. We confirmed the successful establishment of PVR models 3 days after the RPE cell injection. Rabbit eyes at PVR stages between stages 1 and 2 were selected for drug treatment (conducted on day 0). Representative fundus images were recorded to assess the intraocular condition 28 days after the drug treatments. These images revealed complete retinal detachment in the BSS group (stage 5), extensive retinal detachment in the Ripa group (stage 4), and the presence of an intravitreal membrane in the Lipo-Ripa group (stage 1; Fig. 5a). Therefore, the progression of PVR was strongly inhibited in the Ripa group on days 7 and 14, whereas it was markedly suppressed in the Lipo-Ripa group on days 7, 14, 21, and 28, compared with the corresponding data recorded in the control group. These results indicate that Lipo-Ripa exhibited a prolonged and enhanced efficacy for suppressing PVR progression (Fig. 5b). Furthermore, ELISA results demonstrated a marked reduction in the concentrations of periostin, a specific biomarker reflecting PVR status. Periostin is specifically upregulated during PVR development, enhancing the fibrotic properties of RPE cells and thereby accelerating disease progression.^58^^,^^59^ This reduction was observed in vitreous samples acquired from both the Ripa and Lipo-Ripa groups, compared with those collected from the control group. Additionally, Lipo-Ripa exhibited superior efficacy in downregulating periostin levels when compared with Ripa alone (*P* < 0.01; Fig. 5c).

### Toxicity Assessment

To assess the ocular safety of Lipo-Ripa, we conducted in vivo toxicity evaluations in rabbits. IOP was monitored weekly over 4 weeks following a single intravitreal injection of Lipo-Ripa 2 mM into the left eyes of 2 rabbits, with the right eyes injected with BSS serving as controls. IOP values remained within the normal physiological range throughout the observation period in both treated and control eyes; no significant IOP fluctuations were observed in the treated eyes compared to controls (see Supplementary Fig. S3a). Additionally, retinal imaging using the Ret Cam system revealed no abnormal changes in the fundus of Lipo-Ripa–treated eyes (see Supplementary Fig. S3b). Histological examination of whole-eye sections stained with H&E further confirmed no significant difference in retinal morphology and intraocular inflammation between BSS injection and Lipo-Ripa injection (see Supplementary Fig. S3c). These results demonstrate the favorable ocular safety profile of Lipo-Ripa.

## Discussion

In this study, we demonstrated that Lipo-Ripa exhibited enhanced efficacy in suppressing proliferative vitreoretinal diseases, including PVR and advanced nAMD. Through drug screening, we identified ripasudil, a potent ROCK inhibitor, as the most effective agent for inhibiting EMT in RPE cells, as evidenced by upregulated E-cadherin and downregulated α-SMA expression. These findings align with prior studies supporting EMT inhibition as a promising therapeutic strategy for these diseases.^1^^,^^3^

To overcome the limitations of ripasudil's rapid clearance from the vitreous,^24^ we developed a liposomal delivery system using components such as HSPC and DSPE. This formulation (approximately 150 nm diameter) showed excellent physicochemical stability and vitreous retention, making it suitable for intraocular application.^61^^–^^63^ Notably, Lipo-Ripa demonstrated pH-sensitive release, maintaining drug stability at physiological pH while promoting rapid release under acidic conditions mimicking disease microenvironments.

Pharmacokinetic studies demonstrated that Lipo-Ripa substantially reduced passive diffusion across polarized RPE monolayers compared to free ripasudil. This finding indicates that liposomal encapsulation effectively restricts uncontrolled drug leakage, thereby prolonging drug residence within the vitreous. Moreover, partitioning analysis confirmed that nearly all permeating drugs existed in the free form rather than as intact liposomes, suggesting that Lipo-Ripa primarily acts as a local drug reservoir, gradually releasing ripasudil extracellularly while maintaining cellular barriers intact.

Importantly, immunofluorescence analysis revealed that RPE cells internalized fluorescein-labeled liposomes more efficiently than free dye. This enhancement in cellular uptake was particularly significant under pathological conditions, such as in RPE cells stimulated with TGF-β2, which exhibit increased endocytic activity. Thus, liposomal encapsulation not only confers pharmacokinetic stability but also enables disease-targeted drug delivery by exploiting enhanced cellular uptake in fibrotic environments. These observations suggest that under physiological conditions, liposomes primarily serve as localized drug depots. In contrast, in pathological environments, such as PVR or CNV, heightened endocytic activity and acidic conditions facilitate liposome uptake and trigger pH-sensitive drug release.^64^^–^^68^

In vivo, Lipo-Ripa demonstrated markedly improved pharmacokinetics compared to free ripasudil, with higher and more sustained concentrations in both the vitreous and retina. AUC analyses revealed approximately 146-fold and 14.6-fold increases in the vitreous and retina, respectively. The gradual rise in free ripasudil fraction over time (from 69.4% at 24 hours to 92.45% at 120 hours), coupled with the progressive increase in the retina/vitreous partition ratio, confirms that Lipo-Ripa operates as a slow-release depot, providing sustained drug exposure to target tissues.

Therapeutic efficacy studies supported these pharmacokinetic advantages. In a mouse model of laser-induced CNV, Lipo-Ripa treatment significantly reduced subretinal fibrosis compared to BSS and free ripasudil, although CNV size itself remained unchanged. Moreover, Lipo-Ripa more effectively suppressed the ROCK pathway activation in choroidal tissues, indicating superior biological activity at disease sites.

Similarly, in a rabbit PVR model, a single intravitreal injection of Lipo-Ripa markedly attenuated disease progression and periostin levels, maintaining therapeutic effects for at least 28 days. These results demonstrate that liposomal encapsulation extends ripasudil's therapeutic window and enhances its efficacy against intraocular fibrosis.

Importantly, no signs of ocular toxicity, including intraocular pressure elevation, retinal structural damage, or intraocular inflammation, were observed following Lipo-Ripa administration. These findings support the ocular safety of this delivery platform for clinical translation.

Collectively, our findings reveal that liposomal encapsulation enhances the therapeutic profile of ripasudil through multiple complementary mechanisms. By stabilizing ripasudil within the vitreous and effectively restricting passive diffusion, Lipo-Ripa minimizes premature drug loss and preserves high local concentrations. Rather than relying on transcytosis, the formulation enables a controlled extracellular release of free ripasudil, thereby achieving sustained bioavailability within the target tissues. In addition, liposomal encapsulation significantly enhances cellular uptake under pathological conditions, facilitating targeted delivery to fibrotic lesions where therapeutic intervention is most needed. The prolonged vitreous retention of Lipo-Ripa further ensures continuous drug exposure over extended periods, whereas favorable ocular safety profiles confirm its suitability for intravitreal application. These combined properties highlight Lipo-Ripa as a promising platform for the sustained and localized treatment of proliferative vitreoretinal diseases.

Nevertheless, we acknowledge certain limitations. Specifically, this study focused on evaluating liposomes as a delivery platform rather than optimizing clinical intervention timing. Fibrosis represents an irreversible stage in both nAMD and PVR, and early intervention is critical to prevent fibrotic remodeling. Thus, future studies should explore prophylactic or early-stage therapeutic applications, such as combining Lipo-Ripa with anti-VEGF agents during active neovascularization or administering Lipo-Ripa in high-risk patients with PVR before fibrotic membrane formation.

In conclusion, liposomal ripasudil offers a promising approach to achieve sustained and targeted therapy for proliferative vitreoretinal diseases. These findings provide a strong foundation for further preclinical and clinical development of nanoparticle-based therapies in ophthalmology.

## Supplementary Material

Supplement 1
