# Supplementary material for: Liposome Encapsulation Enhances Ripasudil Therapeutic Efficacy Against Proliferative Vitreoretinal Diseases: Implications in Advanced Ocular Treatment
Source: Invest Ophthalmol Vis Sci. 2025 Jun 17;66(6):56. doi: 10.1167/iovs.66.6.56 (PMC12178431; doi:10.1167/iovs.66.6.56)
Supplement: Supplement 1 [file iovs-66-6-56_s001.pdf]

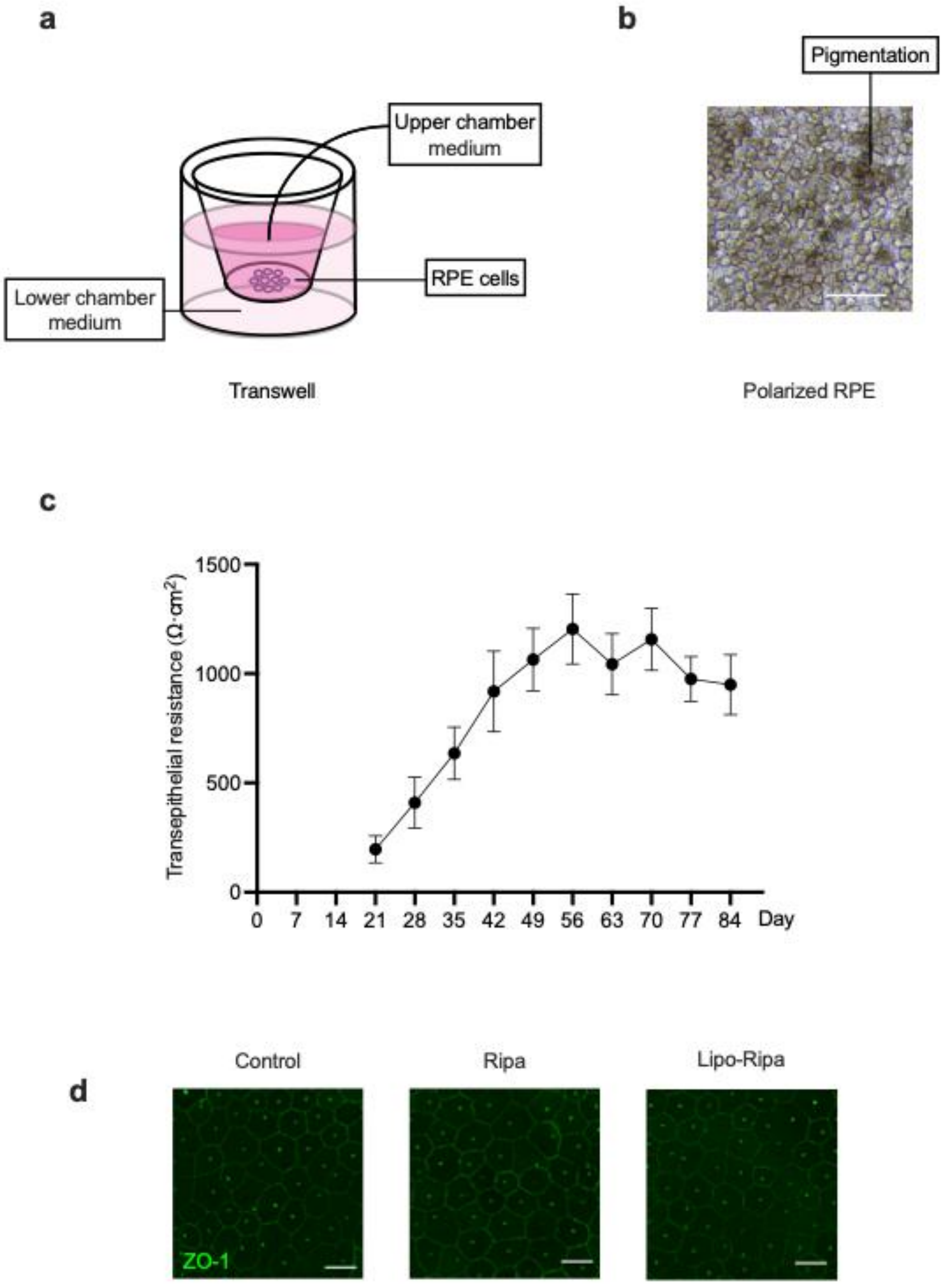

Supplemental Figure 1

Fig. S1. Characteristics of polarized RPE cells

(a) Schematic drawing depicting the transwell system used. (b) RPE cells exhibiting significant pigmentation and a hexagonal mosaic formation after 21 days of incubation in the transwell system. Scale bar = 100 µm. (c) Trend of the transepithelial resistance (TER). (d) Effect of BSS, Ripa, and Lipo-Ripa on immunostaining for ZO-1, a tight junction molecule, in polarized RPE cells. Immunofluorescence show no obvious difference in the groups, bar = 20 µm. RPE, retinal pigment epithelium; BSS, balanced salt solution.

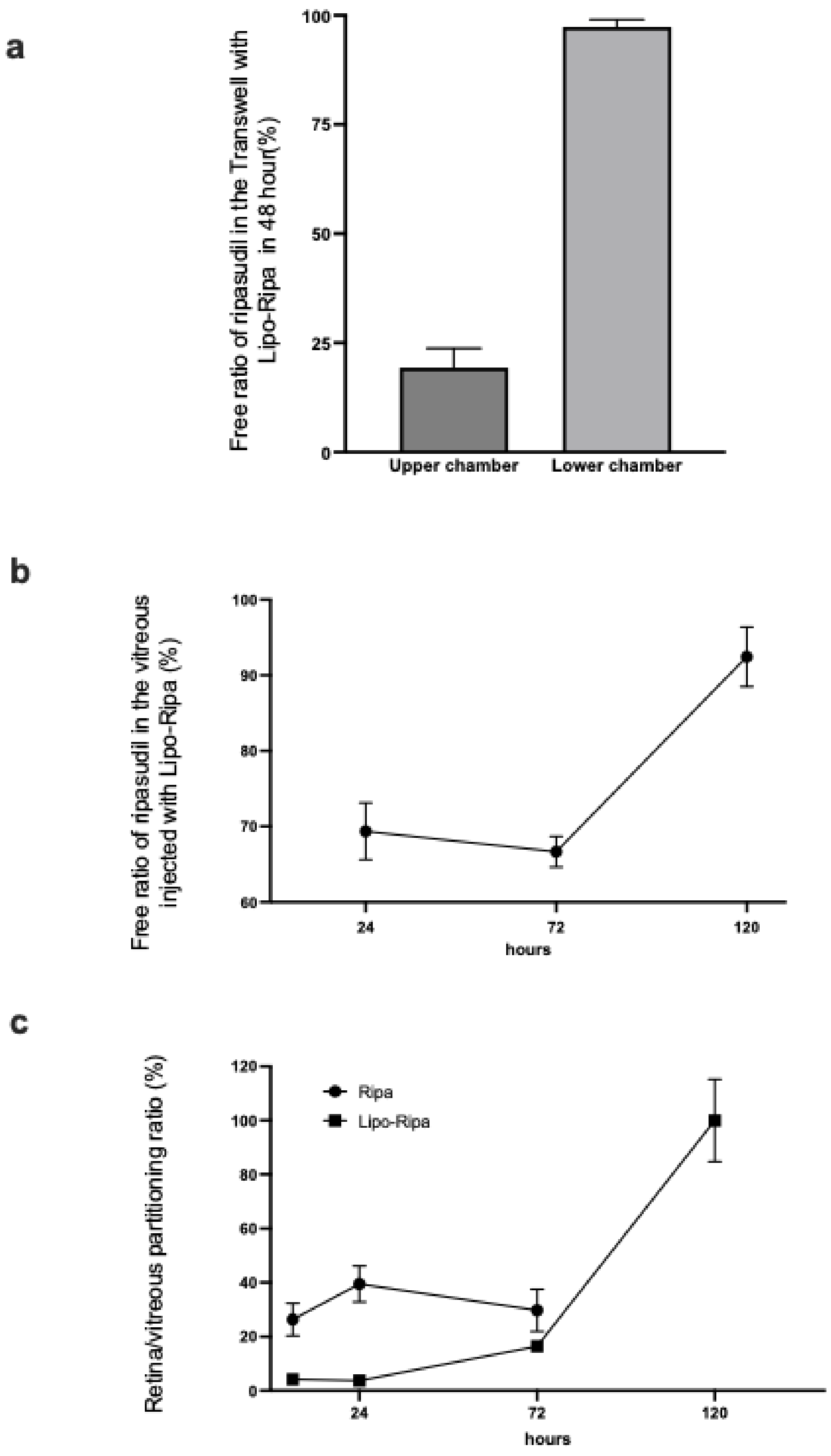

Supplemental Figure 2

Fig. S2. Pharmacokinetic profiling of ripasudil release and tissue distribution

(a) The percentage of free ripasudil relative to the total drug content evaluated at 48 hours post-treatment with Lipo-Ripa. Data are presented as mean ± SEM, n = 3. (b) The percentage of free ripasudil relative to the total drug content in the vitreous evaluated at 24, 72, and 120 hours post-injection of 0.1mL Lipo-Ripa. Data are presented as mean ± SEM, n = 3. (c) Retina/vitreous partitioning ratio of ripasudil following intravitreal injection of 0.1mL of 2mM of Lipo-Ripa or Ripa. Lipo-Ripa. Data are presented as mean ± SEM, n = 3.

a

Day	NO.1 Left	NO.1 Right	NO.2 Left	NO.2 Right
Day0	7.8	7.8	8.0	9.5
Day7	5.8	5.6	6.5	6.6
Day14	8.0	8.1	7.5	6.6
Day21	7.5	8.1	8.6	7.4
Day28	6.3	6.3	7.2	7.0

b

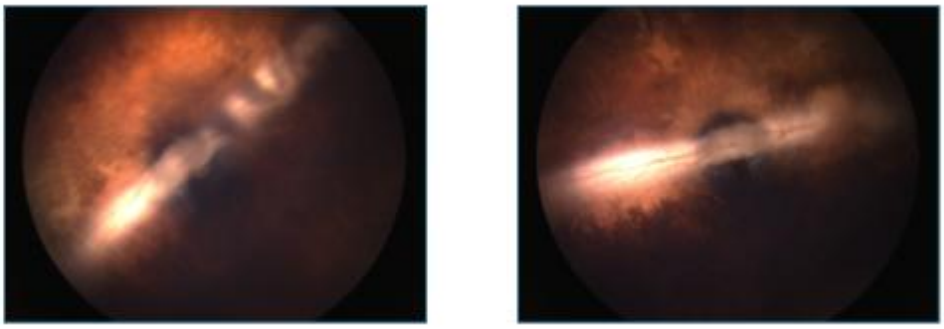

BSS Lipo-Ripa

c

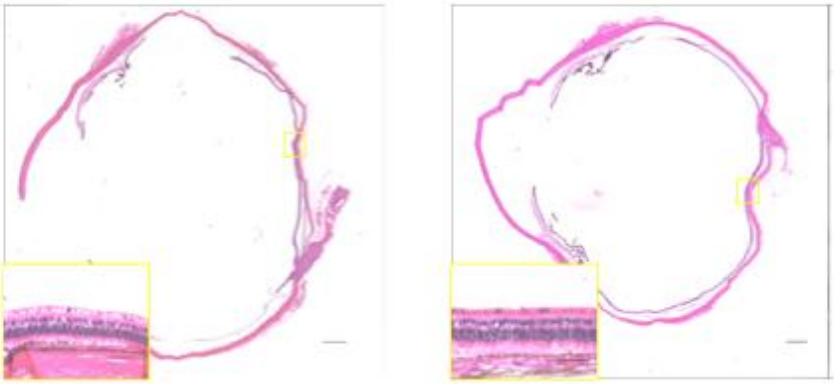

BSS Lipo-Ripa

Supplemental Figure 3

Fig. S3. Toxicity evaluation of Lipo-Ripa.

(a) Intraocular pressure (IOP) was measured weekly over 4 weeks following intravitreal injection of Lipo-Ripa into the left eye of two rabbits, with the right eye injected with BSS serving as a control. (b) Retinal imaging using the RetCam system of control eye and injected with Lipo-Ripa at 2mM. (c) Section of a whole eye stained with hematoxylin and eosin (H&E) in a rabbit injected with Lipo-Ripa at 2mM and BSS as control. Scale bar =1000 μm in main picture. Boxed region in the sections is shown at higher magnification, Scale bar =50 μm.
